# Cervical ultrasound for endotracheal intubation confirmation in dogs by veterinary students: a cadaveric study

**DOI:** 10.29374/2527-2179.bjvm002623

**Published:** 2023-09-05

**Authors:** Bruno Augusto da Silva Rezende, Nicolle Gouvêa Bottoni, Loíse Almeida Cunha, Ângelo Gustavo Novello de Oliveira, Lucas Baptista Motta, Fabio Sartori, Eduardo Butturini de Carvalho

**Affiliations:** 1 Veterinarian. Autonomous, Rio de Janeiro, RJ, Brazil; 2 Undergraduate in Veterinary Medicine, Faculdade de Medicina Veterinária, Universidade de Vassouras. Vassouras, RJ, Brazil.; 3 Veterinarian, MSc. Faculdade de Medicina Veterinária, Universidade de Vassouras. Vassouras, RJ, Brazil.; 4 Veterinarian, DSc. Faculdade de Medicina Veterinária, Universidade de Vassouras. Vassouras, RJ, Brazil.

**Keywords:** canine, esophageal intubation, CPR, cardiopulmonary resuscitation, canino, intubação esofágica, PCR, reanimação cardiopulmonar

## Abstract

Although endotracheal intubation is usually a simple and fast procedure in dogs, some situations can be challenging and lead to the risk of tube misplacement in the esophagus—a life-threatening complication. Hence, confirming intubation is a cornerstone whenever this procedure is performed. Methods such as direct visualization or capnography present limitations insofar as they may be unreliable or unavailable under some circumstances. Ultrasound has emerged as a promising tool to confirm intubation in medicine. However, so far little research has been done on the subject in veterinary medicine. This study’s main goal was to investigate ultrasound performed by veterinary students as a confirmation method for intubation in canine cadavers after a brief training session (25 minutes). A total of 160 exams were performed with a microconvex probe by 20 students in 11 different cadavers on left and right recumbencies. Overall accuracy was 70.6% with a median success rate of 75% and a median time to diagnosis of 25 seconds. The number of correct diagnoses was statistically higher than the wrong ones (p<0.05) without difference between recumbencies. Sensitivity, specificity, and positive and negative predictive values were 72.5%, 68.8%, 69.9%, and 71.4%, respectively. The fastest diagnosis was performed in just 4 seconds, and among the top-performers, one student had 100% accuracy with a mean time to diagnosis of 16.8 seconds, and four students had approximately 88% accuracy. This study showed for the first time that even inexperienced veterinary students can have acceptable accuracy in confirming endotracheal intubation in dogs after a brief training session.

## Resumo

Apesar de a intubação endotraqueal em cães ser frequentemente um procedimento simples e rápido, algumas situações podem ser desafiadoras e levar ao risco de posicionamento da sonda no esôfago - uma grave complicação. Portanto, a confirmação da intubação é uma etapa crucial sempre que o procedimento for realizado. Métodos como visualização direta ou capnografia apresentam limitações e podem ser pouco confiáveis ou indisponíveis sob certas circunstâncias. A ultrassonografia surgiu como uma ferramenta promissora para confirmação da intubação na medicina. Contudo, até o momento pouco foi estudado na veterinária. O objetivo deste estudo foi investigar a ultrassonografia realizada por estudantes de veterinária como método de confirmação para a intubação em cadáveres caninos após um breve treinamento (25 minutos). Foram realizados 160 exames com transdutor microconvexo por 20 estudantes em 11 cadáveres nos decúbitos direito e esquerdo. A acurácia geral foi 70.6% com medianas de taxa de sucesso de 75% e de tempo para diagnóstico de 25 segundos. O número de diagnósticos corretos foi estatisticamente superior aos errados (p<0.05) sem diferença entre decúbitos. Sensibilidade, especificidade, valor preditivo positivo e negativo foram, respectivamente: 72.5%; 68.8%; 69.9% e 71.4%. O diagnóstico mais rápido se deu em 4 segundos e entre os estudantes com melhor performance, um se destacou com 100% de acurácia e tempo médio para diagnóstico de 16.8 segundos enquanto quatro outros obtiveram 88% de acurácia. Este estudo demonstrou pela primeira vez que mesmo estudantes de veterinária inexperientes podem atingir uma acurácia aceitável na confirmação da intubação endotraqueal em cães após um breve treinamento.

## Introduction

Endotracheal (ET) intubation is an essential procedure to maintain open airways in patients. It is routinely performed to ensure effective ventilation, protect lungs from bronchoaspiration, and deliver volatile anesthetics. Albeit usually a simple and fast maneuver in dogs, ET intubation can be quite challenging in some situations such as cardiopulmonary resuscitation (CPR) or trauma, especially for inexperienced veterinarians. Unrecognized ET tube misplacement in the esophagus is a life-threatening complication and a significant source of malpractice claims in medicine (Chenkin et al., 2015; Honardar et al., 2017; Liu et al., 2023). Therefore, confirming correct tube placement is a key step whenever this procedure is performed (Abbasi et al., 2015; Grubb et al., 2020).

Common methods to confirm ET intubation are direct visualization of the tube between arytenoids, condensation inside the tube, and movement of the breathing bag (Grubb et al., 2020). Yet, according to the American Society of Anesthesiologists, the gold standard is the capnograph (Apfelbaum et al., 2022). Two main limitations arise from the use of capnography as a confirmation method for ET intubation. First, capnographs can be expensive and not ubiquitously present in veterinary emergency rooms or even in operating rooms. Secondly, when cardiac output is extremely low—or even zero during cardiac arrest—CO_2_ may be undetectable even if the tube is correctly placed (Gottlieb et al., 2020; Herreria-Bustillo et al., 2016).

Although described decades ago (Raphael & Conard III, 1987), just recently ultrasound (US) has emerged as an alternative to confirm ET intubation in veterinary medicine (Herreria-Bustillo et al., 2016). Studies in humans have shown promising results, demonstrating high accuracy for the method and a fast learning curve (Chenkin et al., 2015; Chou et al., 2011; Liu et al., 2023). Nonetheless, most research has focused on experienced practitioners and up to now only one study was performed in veterinary medicine (Herreria-Bustillo et al., 2016). Thus, it is important to investigate if the method could also aid inexperienced personnel. This study’s main goal was to assess cervical US performed by inexperienced veterinary students as a method to confirm ET intubation in canine cadavers.

## Material and methods

**Figure 1:**
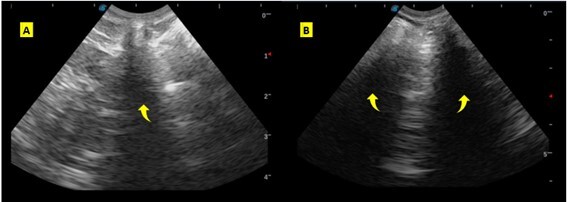
Ultrasonographic transversal view of the ventral cervical region of canine cadaver during: A) endotracheal intubation, showing one reverberation artifact highlighted by the yellow arrow and B) during esophageal intubation, showing two parallel reverberation artifacts (right - tracheal and left - esophageal with an ET tube inserted).

This project has been approved by the ethics committee from the University of Vassouras (5.515.382), where the experiment took place. Canine cadavers—whose deaths had no relationship to the purpose of this study—were donated from the veterinary anatomy department. A total of 11 mixed-breed canine cadavers were unfrozen 24 hours before the experiment. Exclusion criteria were laryngeal, tracheal, or esophageal abnormalities and the presence of lesions in the cervical region. The US equipment used was a Sonoscape S2V with a microconvex transducer (7-9 MHz). ET cuffed tubes were previously chosen as adequate for each cadaver. In order to better simulate a clinical scenario, hair from the cervical region was not clipped and instead of acoustic gel, alcohol was used to help the US exam. In a transversal view, placing the transducer over the ventral cervical region, correct ET intubation is characterized by one hyperechoic image indicating air presence and its’ reverberation artifact being only in the tracheal lumen, whereas an esophageal intubation is characterized by two hyperechoic reverberation artifacts, suggesting the presence of air in both the tracheal and the esophageal lumen, as shown in Figure 1.

Twenty undergraduate students who had already completed the “Image diagnosis” undergraduate course were randomly selected from a list and invited to participate. A pilot study was first conducted with five students (and four cadavers) and a second phase took place six months later with another fifteen students (and seven cadavers) on two different days. A total of 20 students each performing 8 exams resulted in 160 exams. All students engaged in a 25-minute training session before the experiment, consisting in a 10-minute oral presentation by two researchers (B.A.S.R and N.G.B) and a 15-minute hands-on training session with the equipment and one cadaver.

**Figure 2:**
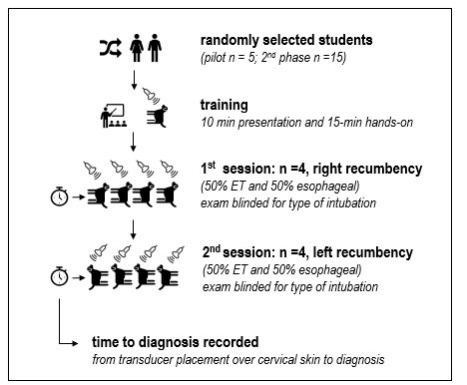
Experimental design. ET - endotracheal; min - minutes; n - sample size.

In a separate room, four canine cadavers were placed side by side on tables. In the first session, cadavers were put in right lateral recumbency and in the second session, in left recumbency. ET or esophageal intubation was randomly performed on each cadaver by one of the researchers (E.B.C). Randomization for the type of intubation was done by flipping a coin in a manner to achieve 50% of ET and 50% of esophageal intubations by the end of the two rounds that each participant took part in. Students were blind to the types and to the total proportion of ET to esophageal intubation in cadavers, and were not allowed to move the cadaver during the exam. Every student participated in two separate rounds, each consisting of four randomly intubated cadavers. They were instructed to perform the exam and give the final diagnostic to one researcher in the room: ET or esophageal intubation. Time was measured using a chronometer from the moment that the student placed the transducer over the cadaver skin to the final diagnosis. Figure 2 summarizes the experimental design.

Statistical analysis was performed in Jamovi 2.3.9. The following combinations were considered as described: ET intubation correctly diagnosed - true positive (TP), esophageal intubation correctly diagnosed - true negative (TN), ET intubation and a diagnosis of esophageal intubation - false negative (FN), and esophageal intubation with a diagnosis of ET intubation - false positive (FP). Accuracy: (TP+TN)/n where n is the sample size; sensitivity: TP/(TP+FN); specificity: TN/(TN+FP); positive predictive value (PPV): TP/(TP+FP); and negative predictive value (NPV): TN/(TN+FN) were calculated with the respective 95% confidence interval (CI95%).

Correct diagnosis proportions were compared between left and right side recumbency by a binomial proportion test (H_0_≠0.5). TP, TN, FP, and FN proportions were compared by the χ^2^ test. Accuracy between students trained by different researchers (B.A.R.S or N.G.B) was compared using the Mann-Whitney test and accuracy between right and left lateral recumbencies with a paired t-test. Values were presented as mean or median followed by the confidence interval of 95% or interquartile range (IQR) depending on their distribution. The significance level was 5% for all tests (α=0.05) and all data was tested for normality using the Shapiro-Wilk test and variance homogeneity using the Levene test.

Sample size was calculated based on the formula n = (z^2^ * S * (1-S)) / E^2^; where n is the total sample size (number of exams), z is the z-score (1.96 for a 95% confidence interval), S the test sensitivity (90% was used as reference based in a conservative approximation of the sensitivity obtained by Herreria-Bustillo et al. (2016)), and E the error margin (0.05). The calculation resulted in 139 exams, and 10% of this number was added considering the possibility of eventual missing values. The final sample size was rounded to 160 exams, which was divided for each of the 20 students.

## Results

A total of 160 exams were performed by veterinary students (40 during the pilot study and 120 in the study’s second phase). Half of the exams were ET and half were esophageal intubations—the same proportion for each lateral recumbency. Each student performed eight different exams, half in each lateral recumbency.

**Figure 3:**
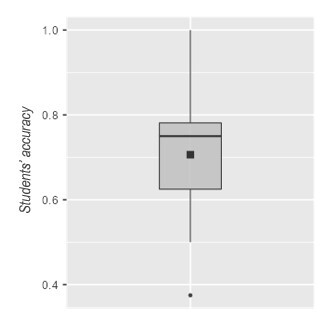
Boxplot of student’s accuracy in diagnosing type of intubation with ultrasound.

Overall accuracy of the method was 70.6% (113 in 160 attempts) with a mean success rate of 5 in 8 attempts for students (median = 75%), as shown in Figure 3. There was no statistical difference in the success rate between students trained by different researchers (p=0.969). The number of correct diagnoses was statistically higher than wrong ones (113 *vs*. 47) in both recumbencies (p<0.05) and there was no difference between accuracy of recumbencies (p=0.562) for the right (68.8% - CI95% 0.581-0.794%) and left (72.5% CI95% 63.3 - 81.7%).

Among 160 exams, there were 58 TP (36.25%), 55 TN (34.4%), 22 FN (13.8%), and 25 FP (15.6%). These values were significantly different (p<0.001; χ^2^ = 27.4). Sensitivity i.e., the ability to detect ET intubations, was 72.5% (CI95% 61.4-81.9%). Meanwhile specificity, i.e., the ability to detect esophageal intubations, was 68.8% (CI95% 57.4-78.7%). PPV, i.e., the probability that an ET intubation diagnosis truly represents a correct intubation, was 69,9%; whereas NPV, i.e., the probability that an esophageal intubation diagnosis truly represents a misplaced tube, was 71.4%. Table 1 summarizes main results.

**Table 1:** Students’ performance in confirming endotracheal intubation with cervical ultrasound.

**Variable**	**Estimate**	**95% Confidence Interval**	
		**Lower**	**Upper**
*Overall accuracy*	70.6%	---	---
*Sensitivity*	72.5%	61.4%	81.9%
*Specificity*	68.8%	57.4%	78.7%
*PPV*	69.9%	---	---
*NPV*	71.4%.	---	---
**Variable**	**Estimate**	**Interquartile range**	
*Mean time to diagnosis*			
*Overall*	25 s	35.3 s	
*Left recumbency*	26.5 s	33.5 s	
*Right recumbency*	23.5 s	34.5 s	

Note: NPV - negative predictive value; PPV - positive predictive value; s - seconds.

**Figure 4:**
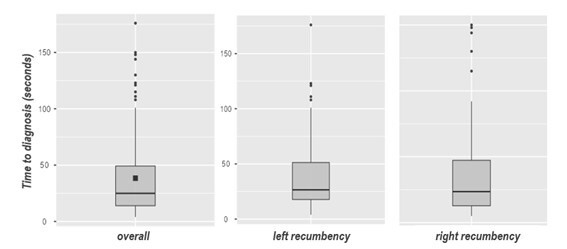
Boxplots of student’s time to diagnosis (overall, left and right recumbency), counting from the moment the transducer touched neck skin to final diagnosis.

Median time to diagnosis was 25 s (IQR 35.3 s) and the distribution was positively skewed (asymmetry = 1.69) as shown by Figure 4. Although the mean time to diagnosis in right lateral recumbency was 2.2 seconds lower than in left recumbency, it was not statistically different (p=0.338). The fastest diagnosis was performed in 4 seconds and the slowest in 176 seconds.

Among top-performing students, it is possible to highlight one with a 100% accuracy and a mean time to diagnosis of 16.8 seconds, and four students with approximately 88% accuracy, all of which had no previous experience in US exams.

## Discussion

Although described in medicine long ago (Raphael & Conard III, 1987), so far only one study investigated US as an intubation confirmation method in dogs. Compared to (Herreria-Bustillo et al., 2016), overall accuracy (70.6% vs. 82.8%), sensitivity (72.5% vs. 91.7%), specificity (68.8% vs. 72.7%), PPV (69.9% vs. 78.6%) and NPV (71.4% vs. 88.9%) were lower in this study. Also, mean time to diagnosis was longer (38.4 vs. 20.2 seconds), despite a positively skewed distribution (1.69) with a median time of 25 seconds—close to the previously reported value. Additionally, there was no difference for time to diagnosis for right or left recumbency, just as (Herreria-Bustillo et al., 2016) demonstrated, suggesting that practitioners could perform the exam on both sides without losing accuracy.

Some factors could be hypothesized as causes to differences observed between this and previous studies. First, the population here studied—veterinary students without prior experience in US—differed from (Herreria-Bustillo et al., 2016), that consisted of practitioners and even one board-certified criticalist. It has been shown that expert emergency physicians achieved higher performance than residents (100% *vs*. 91% sensitivity on thin patients, respectively), indicating that experience may play a central role in performance.

Time of training could also be a hypothesis to explain the lower accuracy observed. In this study a brief training session was offered to students, whereas a similar study provided a longer training session (1 hour) (Herreria-Bustillo et al., 2016). Notwithstanding, the shorter training session could be even worse for inexperienced students in comparison to practitioners. Nevertheless, results here presented—such as accuracy above 70% and top-performers accuracy records as high as 88 to 100%—suggest that the learning curve may not be very long. One study investigated the learning curve of US as a confirmation method. Emergency physicians were able to achieve good performance in interpreting US clips for detecting ET intubation after a brief tutorial and two practice attempts (Chenkin et al., 2015).

On the other hand, the fact that inexperienced students had overall acceptable accuracy indicates that the method can be even more reliable among practitioners with more experience and training. Also, conducting the study with inexperienced students could better reflect reality among Brazilian veterinarians that work in emergency rooms, who are commonly untrained in US.

Also, patients’ characteristics could have impacted the method especially in inexperienced physicians. One study showed that experts had 100% specificity in obese patients while residents had only 48% (Gottlieb et al., 2014). Likewise, small size and unfrozen cadavers could be associated with worse test performance. (Herreria-Bustillo et al., 2016) used fresh cadavers ranging from 10 to more than 32kg while in the present study, cadavers as small as 2kg were used. However, a limitation in this study is that cadavers’ weights were not registered making it impossible to check this hypothesis.

A systematic review found no statistically significant differences in accuracy between US and capnography in confirming ET intubation in neonates (Liu et al., 2023). Yet, considering the brief single training session and inexperienced students performing the exams, results here presented are insufficient to indicate US as a substitute for capnography in ET intubation confirmation. Further research should aim for longer testing or repeated training sessions and assessing the method’s accuracy in live patients. The considerable amount of FP (false positives) leaves space to a critical situation: approximately 16 out of 100 patients with a misplaced tube would have received an ET intubation diagnosis. This is a situation that could lead to death and malpractice claims.

Compared to the present results, a noticeable lower time to diagnosis—as low as 3 seconds—has been observed in human studies (Abbasi et al., 2015; Chou et al., 2011; Gottlieb et al., 2019; Muslu et al., 2011). It is reasonable to suggest that all above-mentioned hypotheses—such as time of training, operators’ experience levels, patients’ or cadavers’ characteristics—could also be applied here. A curvilinear probe—used in the present research—could raise exam time, as shown by (Gottlieb et al., 2019).

Last but not least, it is worth noting that a group of students with no previous experience with US had excellent performances. One student had 100% accuracy with a mean time to diagnosis of less than 17 seconds and four other students had approximately 88% accuracy, suggesting that even with inexperienced operators under little training, the method can be highly effective.

## Conclusions

US can be a promising tool to help ET intubation confirmation when other methods are unavailable or ineffective. Despite showing lower accuracy than previous studies in humans and the only study in dogs, this study has demonstrated that after brief training, inexperienced students can have acceptable performances. Further studies should focus on investigating the hypothesis that US is a reliable method for ET intubation confirmation in live patients when performed by veterinarians with more solid training.
